# Ocean acidification modifies biomolecule composition in organic matter through complex interactions

**DOI:** 10.1038/s41598-020-77645-3

**Published:** 2020-11-26

**Authors:** Julia Grosse, Sonja Endres, Anja Engel

**Affiliations:** Department of Biological Oceanography, GEOMAR Helmholtz Centre for Ocean Research Kiel, Kiel, Germany

**Keywords:** Biogeochemistry, Ocean sciences, Environmental microbiology

## Abstract

The main source of marine organic carbon (OC) is autotrophic production, while heterotrophic degradation is its main sink. Increased anthropogenic CO_2_ release leads to ocean acidification and is expected to alter phytoplankton community composition, primary production rates and bacterial degradation processes in the coming decades with potential consequences for dissolved and particulate OC concentration and composition. Here we investigate effects of increased *p*CO_2_ on dissolved and particulate amino acids (AA) and carbohydrates (CHO), in arctic and sub-arctic planktonic communities in two large-scale mesocosm experiments. Dissolved AA concentrations responded to *p*CO_2_/pH changes during early bloom phases but did not show many changes after nutrient addition. A clear positive correlation in particulate AA was detected in post-bloom phases. Direct responses in CHO concentrations to changing *p*CO_2_/pH were lacking, suggesting that observed changes were rather indirect and dependent on the phytoplankton community composition. The relative composition of AA and CHO did not change as a direct consequence of *p*CO_2_ increase. Changes between bloom phases were associated with the prevailing nutrient status. Our results suggest that biomolecule composition will change under future ocean conditions but responses are highly complex, and seem to be dependent on many factors including bloom phase and sampling site.

## Introduction

The increase in atmospheric carbon dioxide (CO_2_) concentration due to anthropogenic emissions is changing the ocean’s carbonate chemistry^1^, leading to ocean acidification. As a consequence, the pH of ocean waters has decreased by about 0.1 units since 1900 and is predicted to drop by another 0.3–0.5 units until 2100, with Arctic waters being impacted highest^2^. Ocean acidification promotes the uptake of inorganic carbon by phytoplankton leading to higher primary production rates, and can subsequently result in an increased release of dissolved organic matter (DOM)^3–5^. The molecular composition of marine DOM is highly complex, changes with depth, season, and region^6^, and only a few compounds can be identified in terms of structure by chemical analysis. These compounds include labile and semi-labile components, such as carbohydrates (CHO), amino acids (AA), proteins, peptides, lipids, and nucleic acids, all of which are enriched in freshly produced DOM^6–8^. In total, up to 90% of the DOM produced during photosynthesis is degraded by heterotrophic bacteria in the water column, transformed into bacterial biomass or respired to inorganic carbon which potentially feeds back in atmospheric CO_2_ concentrations^9–11^.

Studies investigating the effects of increasing *p*CO_2_/decreasing pH on DOM dynamics show a wide range of responses. In several studies no significant changes were detected in dissolved organic carbon (DOC) and other DOM compound concentrations^12–14^. Some studies found significant changes in DOC concentration but the responses were opposite. While two studies in the Arctic and Finland, led by Engel et al. and Paul et al., detected DOC accumulation under very high *p*CO_2_ levels^3,15^, Yoshimura et al.^16^ detected increased DOC removal rates under increased CO_2_ in the Sea of Okhotsk.

AA account for a small fraction (of 1–3%) of the ocean DOC pool^6^, but they are essential for growth of higher organisms and can either be directly incorporated into the biomass or be used as pre-cursors for the synthesis of more complex AA^17,18^. CHO contribute between 10 and 25% to the DOC pool^6^ and are also a primary energy source for heterotrophic bacteria^19^. Additionally, dissolved AA and CHO are primary building blocks for the formation of more complex marine gel-like particles, an important fraction of the particulate OM pool in the ocean^20^. These particles provide a surface for bacteria to attach and grow, and can become hotspots of microbial degradation^21,22^ while also adsorbing large amounts of nutrients^23,24^ and fostering export of organic and inorganic matter^20^. Two forms of gel-like particles can be distinguished by staining methods: (1) Transparent exopolymer particles (TEP) are rich in CHO and are stained with Alcian Blue^21^ and (2) Commassie Stainable Particles (CSP) are stained with Commassie Blue and are characterized by a higher content of AA^23^.

Ocean acidification may also affect formation and concentration of these gel-like particles and hence the AA and CHO signal in the total or particulate AA and CHO fraction. The amount of TEP was shown to increase with *p*CO_2_ in studies by Endres et al. and MacGilchrist^25,26^. Another study found AA accumulation was slightly decreased at elevated *p*CO_2_^12^, but investigations into CSP changes have not yet occurred. While some efforts were made to determine effects of ocean acidification on gel-like particles, there is no study, to our knowledge, that has investigated compositional changes of individual AA and CHO in plankton communities exposed to ocean acidification.

Here we show results from two large off-shore mesocosm campaigns conducted in Arctic Kongsfjorden, Svalbard Archipelago, Norway in 2010^27^ and Sub-arctic Raunefjord near Bergen, Norway in 2011^28^. The Svalbard study manipulated nutrient-poor, post-bloom fjord water dominated by haptophytes and prasinophytes with only minor contributions of diatoms. There the *p*CO_2_ levels were increased from ~ 180 to 270–1420 µatm^27^. The Bergen community was dominated by diatoms, with some chlorophytes, cryptophytes and haptophytes, reflecting a composition more common during the spring bloom. There *p*CO_2_ levels were increased from 310 to 395–3045 µatm^28^.

For both studies, we investigated changes of AA and CHO composition in the natural plankton communities as a response to *p*CO_2_-induced changes in pH. We hypothesize that *p*CO_2_ increase will affect the production and subsequent release of AA and CHO by phytoplankton and leave a measurable trace in the water-column. We expect to see changes in absolute pool concentrations as well as in the compound specific composition of the different pools. The changes will differ between bloom phases and nutrient availability (before and after nutrient addition).

Both campaigns were separated into two stages; during the first stage *p*CO_2_ levels were manipulated, while during a second phase all mesocosms additionally received a resupply of inorganic nutrients. Based on chlorophyll* a* development during both campaigns other publications identified distinct bloom-phases within the two stages, which we continued to use for the analysis of our data^27,28^. A first phytoplankton bloom developed after CO_2_ addition during both campaigns. A second bloom developed after nutrients addition, about halfway through the sampling period. A third phytoplankton bloom developed in Kongsfjorden/Svalbard. Clear post-bloom phases were identified in the Raunefjord/Bergen (low phytoplankton biomass after depletion of at least one inorganic nutrient).

## Results

### Organic matter development during bloom phases

**Figure 1:**
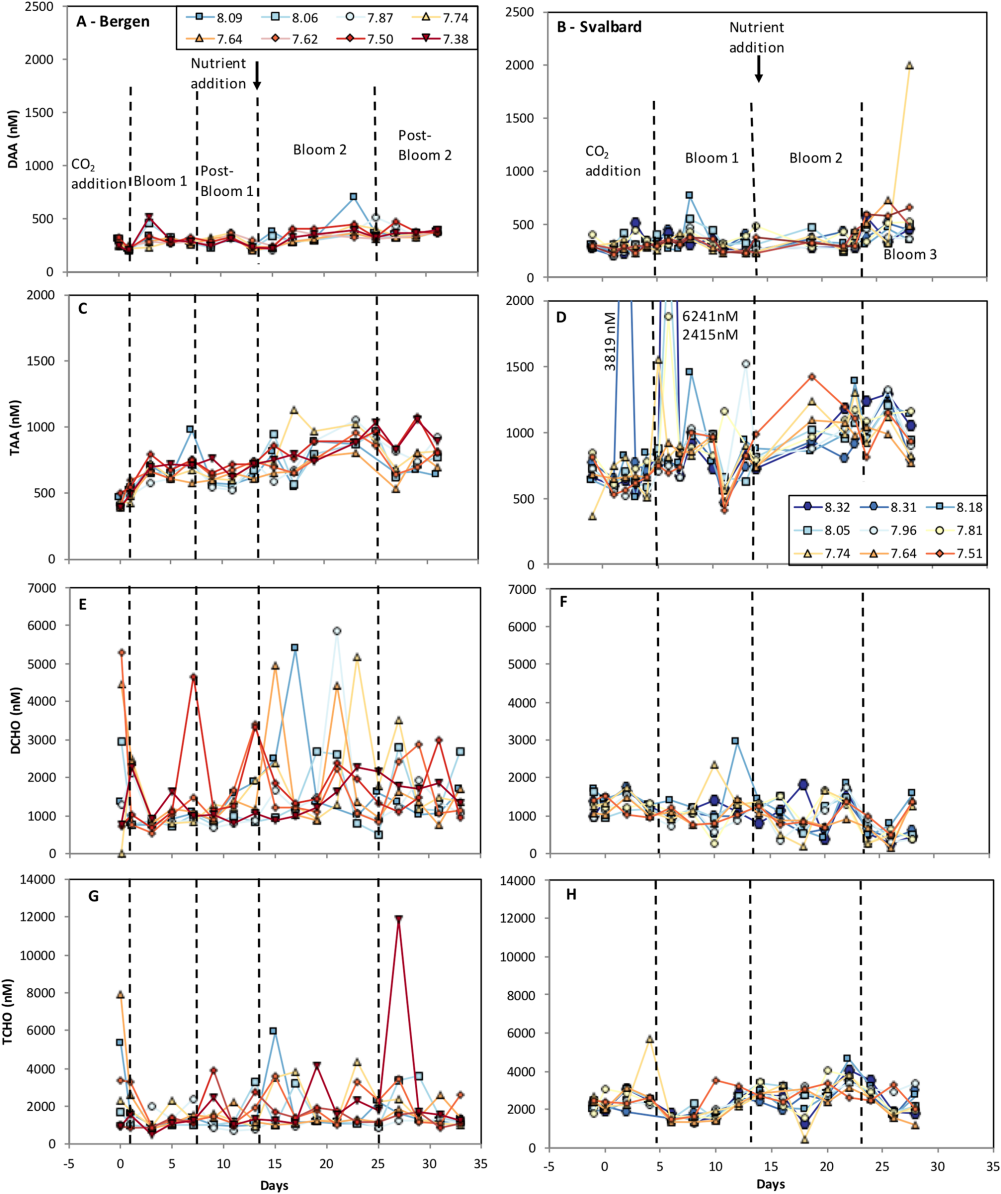
Development of concentrations of dissolved and total amino acids (**A**–**D**) and dissolved and total carbohydrates (**E**–**H**) for all mesocosms in Raunefjord/Bergen and Kongsfjorden/Svalbard. Numbers in legend refer to initial pH values of respective mesocosm, also see Table 1.

Development of AA and CHO concentrations (sum of individually measured compounds) in the dissolved (DAA/DCHO) and total fractions (TAA/TCHO) were illustrated in Fig. 1. AA concentrations ranged between 198 and 1998 nM DAA (n = 315) and 365–6241 nM TAA (n = 313) across both campaigns and all bloom phases (including CO_2_ addition phase). DAA contributed to TAA with 42 ± 11% (average ± stdev, n = 311, Fig. 1). The remaining 58 ± 11% (n = 311) are hereafter referred to as particulate AA (PAA).

**Table 1:** Initial values of *p*CO_2_ and pH in all mesocosms at day t_5_ (Bergen) and t_8_ (Svalbard) when equilibrium of added CO_2_ with dead space was reached.

**Raunefjord, Bergen**											
pCO_2_			310	395	590		890	1165	1425	2060	3045
pH			8.13	8.04	7.88		7.71	7.60	7.52	7.36	7.20
											
**Kongsfjorden, Svalbard**											
pCO_2_	185	185	270	375	480	685	820	1050	1420		
pH	8.32	8.31	8.18	8.05	7.96	7.81	7.74	7.64	7.51		
											

**Figure 2:**
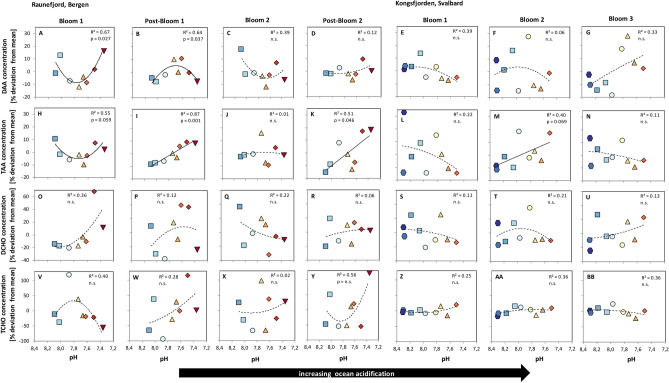
DAA, TAA, DCHO and TCHO concentrations (%deviation from mean) as a function of decreasing pH (total scale; increasing *p*CO_2_). Linear and binomial regressions were performed and significance of trends is stated in each plot. Symbols indicate deviation from mean of all mesocosms over time in different phases of bloom development for both mesocosm campaigns. For symbol identification see Table 1. The bloom phases and time point of nutrient additions were identified by Schulz et al.^27,28^. Dashed lines indicate non-significant relationships. Nutrient additions occurred at the end of Post-Bloom 1 (Raunefjord/Bergen) and between Bloom 1 and Bloom 2 (Kongsfjorden/Svalbard).

Same shapes and colors indicate similar levels of ocean acidification reached between campaigns. Symbols and color code denote those in Figs. 1, 2 and S2.

CHO concentrations ranged between 153 and 5846 nM DCHO (n = 294) and 477–11,882 nM TCHO (n = 279), including all measurements. The contribution of DCHO to TCHO varied greatly within and between experiments and reached 70 ± 24% (n = 87, range 15–100%) and 45 ± 19% (n = 146, range 9–93%) in the Raunefjord/Bergen and Kongsfjorden/Svalbard campaign, respectively. Contrary to AA, particulate CHO could not be calculated for many sampling points (see “Material and methods” section for detailed information).

Overall, large inter-daily variations in the compound class concentrations made it difficult to detect responses of the individual mesocosm to changes in pH based on the concentration data alone. We therefore used an approach to determine the deviation of each mesocosm from the daily average across all mesocosms to identify potential effects of pH on bulk organic matter constituents. Afterwards, all values from one bloom-phase were averaged for each mesocosm. Figure 2 gives an overview of the results for total DAA, TAA, DCHO and TCHO (sum of individually measured compounds) using this approach. DAA and TAA showed significant correlations with pH for several bloom and post-bloom phases while no significant relationships were found in DCHO and TCHO. Additionally, responses were found for many individual AA and also for several CHO.

### Changes of individual AA

**Figure 3:**
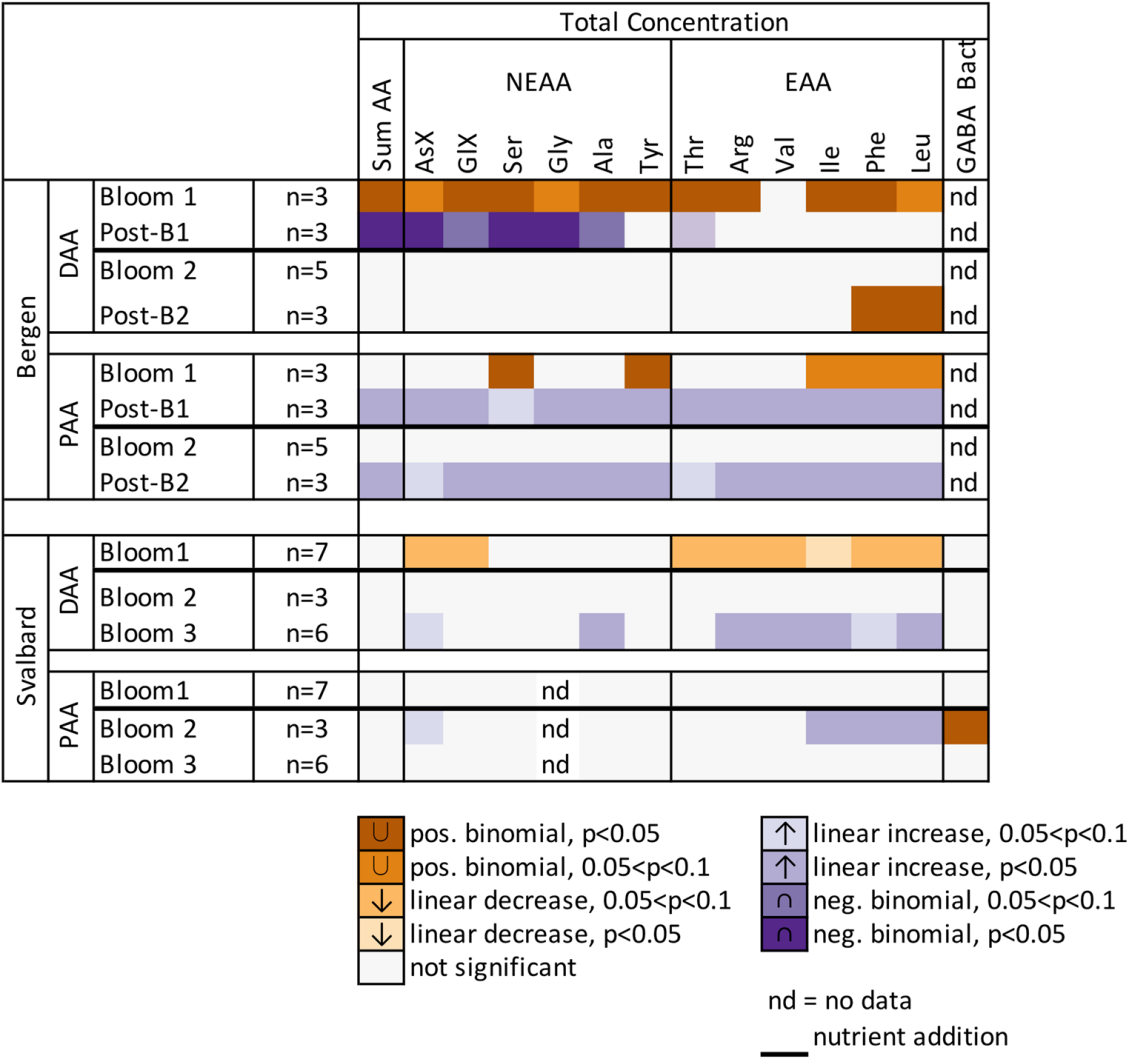
Heat-map summarizes significance levels for all individual AA (DAA and PAA) as calculated in Fig. 1. Bloom phase-averaged response in mean deviation of concentrations against decreasing pH. Refer to Table 2 for acronyms.

Significant correlations of MD with pH were found for several individual AA across both campaigns, in DAA and PAA and during several bloom stages (Fig. 3). For example, during Bloom 1 in Raunefjord all DAA (except Val) were correlated with pH in a positive binomial function. This means that AA concentrations decreased with decreasing pH down to 7.72–7.60 (*p*CO_2_ 890–1165 µatm), before concentrations increased again with pH < 7.60 (*p*CO_2_ > 1165 µatm, e.g. Fig. 2A). During Post-Bloom 1 this picture reversed and concentrations of DAA and non-essential AA increased in mesocosms with pH down to 7.72–7.60, and decreased in mesocosms with pH < 7.60. Changes in PAA were mainly observed during Post-Bloom phases (Bergen) and were only occasionally found in the Svalbard campaign. PAA during Bloom 1 (Raunefjord/Bergen) showed significant correlations with pH for the AA Ser, Tyr, Leu, Ile and Phe using a positive binomial function, again with a lowest MD for mesocosms with pH 7.72–7.60 (*p*CO_2_ to 890–1165 µatm). During Post-Bloom 1 (Raunefjord/Bergen) all individual PAA showed significant increases with decreasing pH.

**Table 2:** Glossary of abbreviations of sub-classes and individual amino acids and carbohydrates.

Amino acids		Carbohydrates	
DAA	Dissolved amino acids	DCHO	Dissolved carbohydrates
PAA	Particulate amino acids	TCHO	Total carbohydrates
TAA	Total amino acids	Fuc	Fucose
NEAA	Non-essential amino acids	Rha	Rhamnose
EAA	Essential amino acids	Ara	Arabinose
AsX	Asparagine + aspartic acid	Gal	Galactose
GlX	Glutamine + glutamic acid	Glc	Glucose
Ser	Serine	ManXyl	Mannose/xylose
Gly	Glycine	GalN	Galactosamine
Ala	Alanine	GlcN	Glucosamine
Thr	Threonine	GlcA	Gluconic acid
Tyr	Tyrosine	GalUA	Galacturonic acid
Arg	Arginine	GlcUA	Glucuronic acid
Val	Valine	MurA	Muramic acid
Ile	Isoleucine		
Phe	Phenylalanine		
Leu	Leucine		
GABA	γ-Aminobutyric acid		

In Kongsfjorden/Svalbard during Bloom 1 significant negative correlations were found with decreasing pH for AsX, GlX and all essential AA in the DAA fraction, while no significant relationships were found for PAA (Fig. 3).

A few correlations of MD with *p*CO_2_ were also found after nutrient addition. However, they were limited to a few individual compounds such as DAA Phe and Leu in Post-Bloom 2 (Raunefjord/Bergen) and DAA AsX, Ala and multiple essential DAA during Bloom 3 in Kongsfjorden/Svalbard. Within the PAA fraction MD correlated positively with decreasing *p*CO_2_ for all AA during Post-Bloom 2 (Raunefjord/Bergen) and but only for AsX, Phe, Ile and Leu during Bloom 2 (Kongsfjorden/Svalbard).

**Figure 4:**
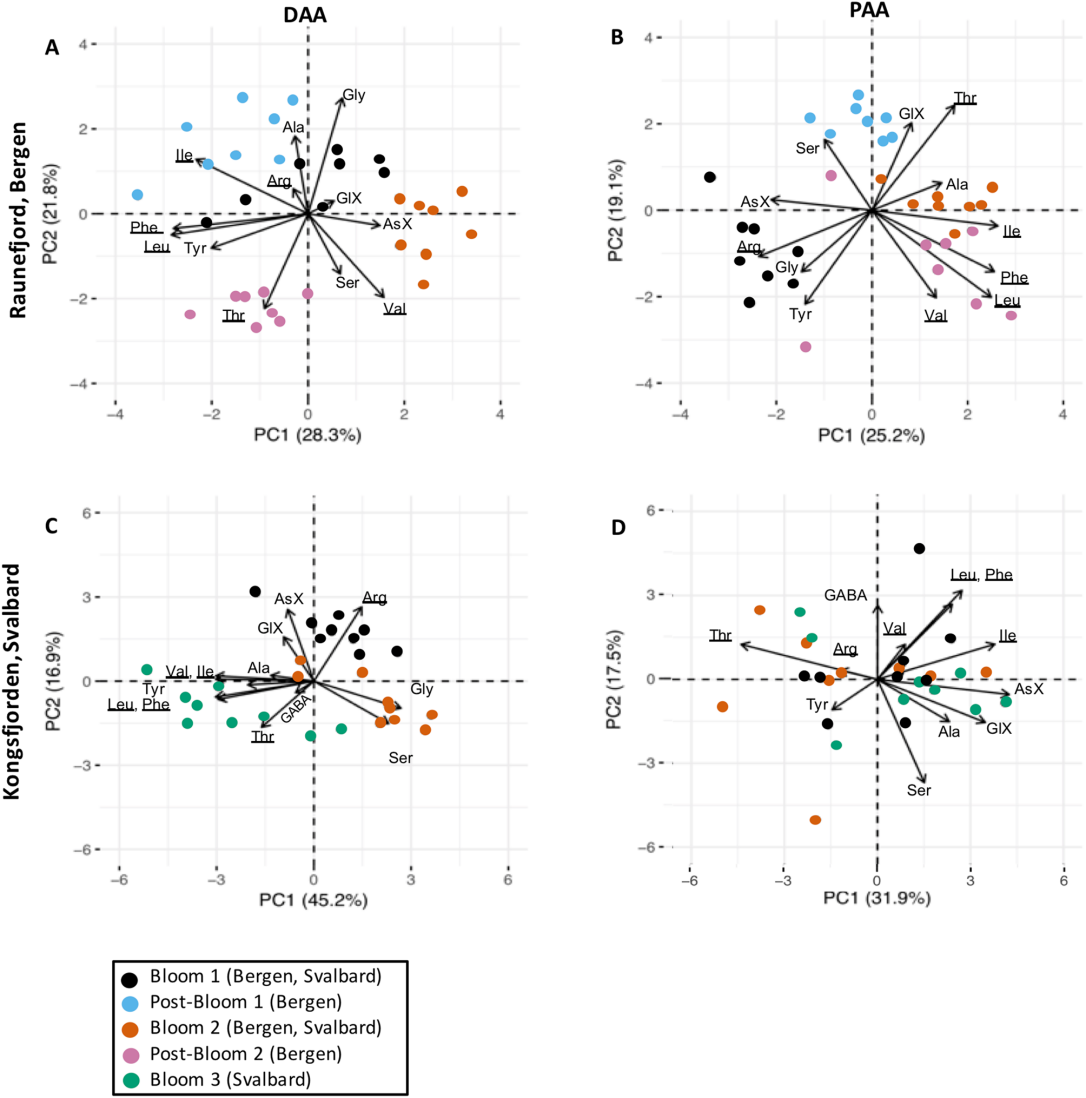
PCA biplot of relative contribution of individual AA to total DAA (**A**) and PAA (**B**) concentration in Raunefjord/Bergen and total DAA (**C**) and PAA (**D**) in Kongsfjorden/Svalbard. The AA GABA was excluded from (**A**,**B**) and Gly from (**D**) (see “Material and methods” section for details). Refer to Table 2 for acronyms. Symbols color represents bloom stage. AA essential to higher trophic levels are underlined.

Relative changes (mol%) in AA composition were evaluated using principal component analysis (PCA) for individual AA of DAA and PAA of Bergen and Svalbard experiments, respectively. The first two components (PC1, PC2) explained 44–62% of the variance in the dataset (Fig. 4). The composition of DAA changed between experimental phases in both campaigns (Fig. 4A,C). In Raunefjord/Bergen the DAA composition during Post-Bloom 1 was enriched in Ala, Ile and Arg, during Bloom 2 enrichment in AsX was seen and during Post-Bloom 2 Thr was enriched. DAA in Kongsfjorden/Svalbard were enriched in AsX, GlX and Arg during Bloom 1. After nutrient addition, Gly and Ser seemed to be enriched in some mesocosms during Bloom 2 (Fig. 4C). Bloom 3 showed enrichment in the essential AA Val, Ile, Leu, Phe and Thr, as well as non-essential AA Tyr. Changes in the relative composition of PAA were only found in Raunefjord/Bergen (Fig. 4B). There, Bloom 1 showed enrichment in AsX, Arg, Gly and Tyr while Post-Bloom 1 showed enrichment in Ser and GlX. After nutrient addition, during Bloom 2 and Post-Bloom 2, data points cluster together and show enrichment in Ala, and the essential AA Ile, Leu, Phe and Val.

### Changes in CHO pools

**Figure 5:**
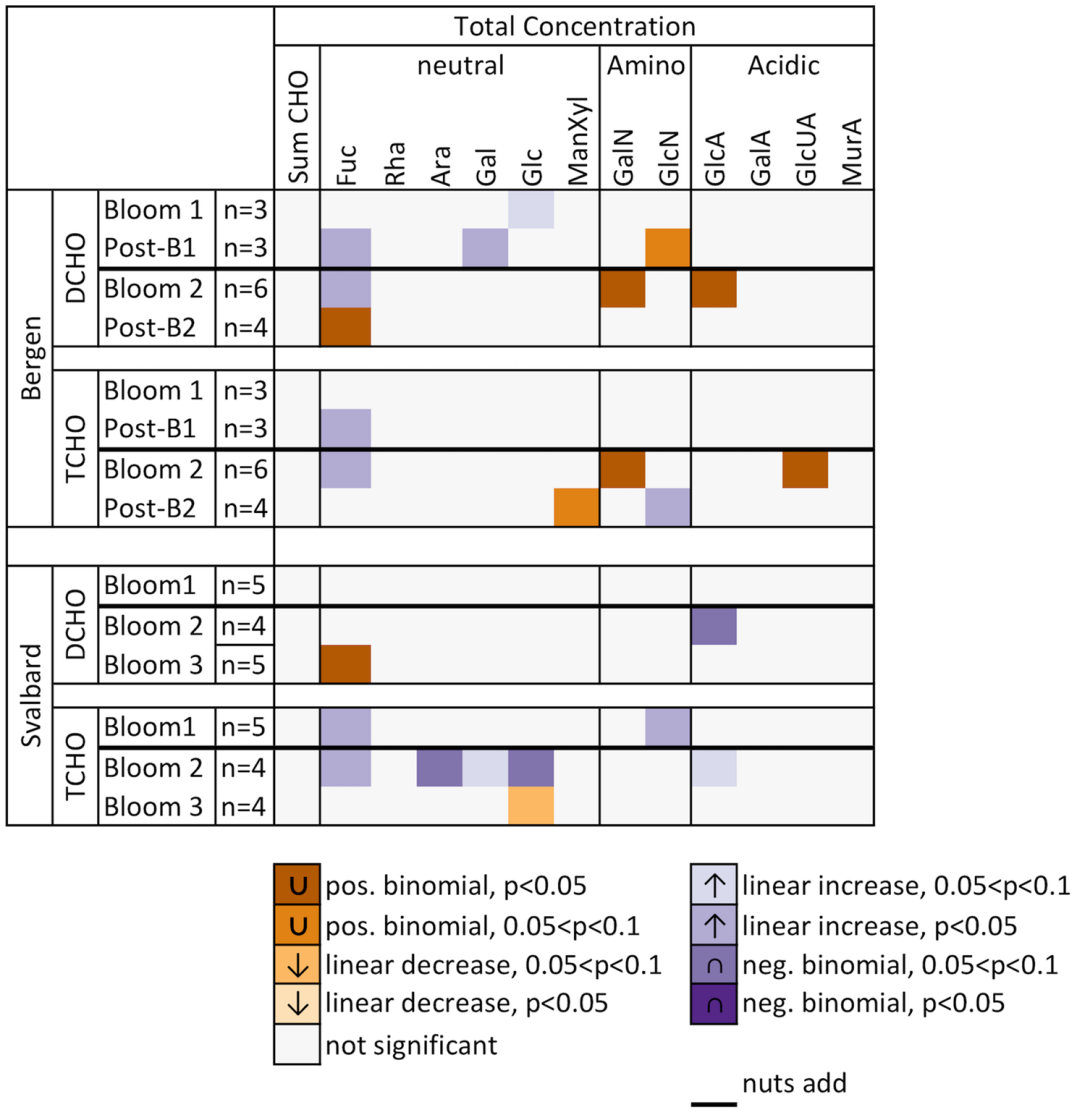
Heat-map shows bloom phase-averaged response in mean deviation of concentrations of individual dissolved and total carbohydrates (DCHO, TCHO) with decreasing pH. Refer to Table 2 for acronyms.

Changes in the CHO pools were much less pronounced compared to AA and were restricted to a few individual compounds (Fig. 5). For example, in Raunefjord/Bergen only Glc showed significant positive correlation with decreasing pH during Bloom 1 and only Fuc, Gal and GlcN showed significant correlations with decreasing pH during Post-Bloom 1. Similarly, for Kongsfjorden/Svalbard only two CHO showed significant correlations within the DCHO during the entire experiments, namely, GlcA during Bloom 2 and Fuc during Bloom 3. Furthermore, significantly correlations for TCHO did also not show a clear pattern.

**Figure 6:**
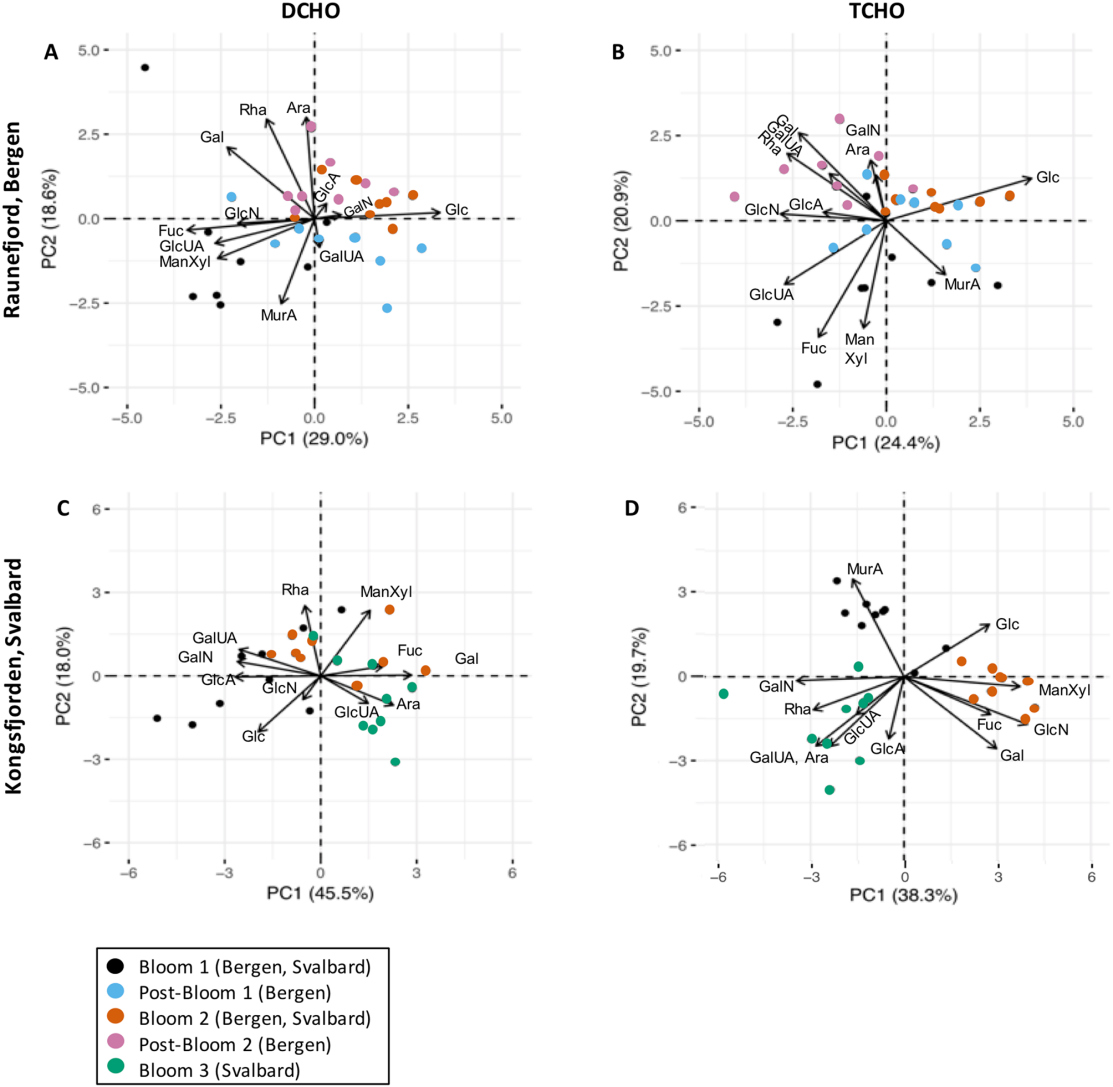
PCA biplot of relative contribution of individual CHO to total DCHO (**A**) and TCHO (**B**) concentration in Raunefjord/Bergen and total DCHO (**C**) and TCHO (**D**) in Kongsfjorden/Svalbard Experiment. Refer to Table 2 for acronyms. Symbols color represents bloom stage.

However, considering relative changes (mol%) in CHO composition using PCA showed clear differences between (post-) bloom phases (Fig. 6). The first two components of these PCAs explained 45–64% of the variation (Fig. 6). In Raunefjord/Bergen, both DCHO and TCHO, had higher relative contributions of Fuc, Man/Xyl, GlcUA and MurA before nutrient addition. During Post-Bloom 2, the mesocosms formed a defined group that was enriched especially in neutral TCHO Rha and Gal as well as GalA (Fig. 6A,B).

In Kongsfjorden/Svalbard the DCHO pool was enriched in GalA, GalN, GlcA and Glc before nutrient addition, indicating that gel-like particles in the form of TEP may have been present in detectable forms (Fig. 6C). The TCHO pool in Kongsfjorden/Svalbard showed three distinct clusters. MurA was relatively enriched before nutrient additions, while after nutrient addition Gal, Fuc, Man/Xyl and GlcN showed enrichment during Bloom 2 and Rha, Ara, GalA, GlcA, GalN and GlcUA showed relative enrichment during Bloom 3 (Fig. 6D).

## Discussion

Ocean acidification triggers increased primary production and DOC release in phytoplankton, as well as bacterial growth and respiration^3,29–31^. Investigations on semi-labile compounds such as AA and CHO however are currently scarce. During two mesocosm campaigns we determined the responses of AA and CHO quantity and quality (composition of individual compounds) as a response to CO_2_ addition. The quantity of AA and CHO is controlled by biomass production and their subsequent release into the DOC pool^32,33^. Increased rates of primary production and DOC release have previously been described for the Kongsfjord/Svalbard^3^ and is reflected in this study by increased contributions of DAA to DOC and DON during Bloom 2 in the Kongsfjorden/Svalbard experiment (Fig. S2). There were no significant differences in the contributions of DCHO and DAA to the total pool of DOC and the contributions of DAA to total DON for the majority of bloom phases. However, a more detailed look on the level of individual compounds revealed complex changes within the AA and CHO pools with some individual DAA seeming to be most affected by pH changes.

Overall, the total concentrations of DAA and DCHO measured in our study are within the range of other studies done in Arctic Seas^7,34,35^. CO_2_ dependent changes in AA and CHO seemed to be decoupled, lacking synchronized changes between the AA and CHO fractions. When AA concentrations are affected (depending on bloom-phase and fraction) most or even all individual AA were either increasing or decreasing. On the other hand, response in CHO was limited to a few individual compounds only. All investigated AA are involved in protein synthesis (except GABA), hence need to be produced in relative proportions. Therefore, AA concentrations and composition in phytoplankton occur within a rather narrow margin across phytoplankton groups^36^. On the contrary, the composition of individual CHO to the total CHO pool varies greatly between different groups of phytoplankton^37^.

Another factor affecting relative changes in AA and CHO composition is nutrient availability. In our study, changes in %mol occurred between bloom-phases especially in AA (Figs. 4, 6). These qualitative changes can be linked to nutrient availability or changes in community composition^32,38^. For example, N-availability affects the synthesis of EAA such as Phe, Ile, Leu and Val, because they require longer synthesis steps than NEAA. These additional synthesis steps in return require additional enzymes, proteins themselves, and down-regulation of certain amino acids during N scarcity limits N requirement of cells^38^. Usually, EAA cluster in PCA plots (Fig. 4, underlined compounds) and their contribution increases after nutrient addition. Furthermore, an increased contribution of GlX often indicates exponential bacterial growth^39^ and different bacterial communities can take up individual DAA preferentially^40^. CHO composition differs between phytoplankton groups, e.g. released DOC from the diatom *Skeletonema costatum* shows high contributions of Fuc, while haptophytes release larger relative amounts of Gal, Xyl and Ara^41^. MurA is uniquely found in bacterial cell walls and can be used to identify increased contributions of bacteria^42^, while nutrient limitation increases the synthesis of storage CHO such as glycan which is rich in Glc^43^. Overall, the dynamic changes in AA and CHO in organic matter are complex and emerging patterns seem to depend e.g. on site, nutrient availability, phytoplankton community structure, bacterial activity and likely additional factors.

*p*CO_2_/pH dependent trends in AA dynamics seemed much clearer in the Bergen campaign, with similar trends observed in the Svalbard campaign for a more limited set of AA. A direct effect of CO_2_ additions during the first bloom-phase was the decrease in DAA concentrations with decreasing pH. Nutrient additions seem to have no direct effect on DAA during the second bloom. In Bergen, clear post-bloom phases existed and illustrated a reversed trend compared to the bloom phases. There DAA concentrations increased with decreasing pH demonstrating the release of labile DOM when bloom biomass was decaying. One could argue that it is imperative to distinguish between bloom and post-bloom phases to detect the described patterns, which should be considered in future studies, otherwise the opposite patterns may cancel each other out.

The two mesocosm campaigns were comparable in experimental design, length of study and the parameters sampled. However, the Raunefjord/Bergen campaign covered a much larger range of pH/*p*CO_2_ treatments, including three mesocosms that were beyond the pH predicted for 2100^28^. Deppeler et al.^44^ found that under high *p*CO_2_ (between 953 and 1140 µatm) the photosynthetic health of phytoplankton cells was negatively affected resulting in lower gross primary production. Lower primary production rates would subsequently result in a diminished DOC and DAA release. To investigate if such as tipping point was also present in the Raunefjorden/Bergen mesocosms we applied a binomial fit to the data. We found that Bergen DAA concentrations displayed such as tipping point at a pH between 7.72 and 7.60 (*p*CO_2_ 890–1165 µatm). At this threshold the phytoplankton tolerance to Ocean Acidification seems to have reached a point at which DAA release was not further increased with lower pH but reversed instead. Including the very high *p*CO_2_ mesocosms in Bergen confirms this tipping point at a similar pH/*p*CO_2_ range. This needs to be taken into consideration when planning future studies and evaluating data trends. Additionally, the species composition of the phytoplankton community can further affect results and is needs to be considered. Some species (even within the same genus, e.g. *Navicula*) are better adapted to lower pH because they experience natural fluctuations over the annual cycle^44,45^. Fluctuations are especially large in polar waters where CO_2_ builds up under the sea ice in winter which is linked to respiratory processes due to a lack of light supporting primary production. In spring a rapid decrease in CO_2_ is linked to the spring bloom, and can cause an annual variation in pH of 0.8 units^45^.

The two studies presented here differed in their initial phytoplankton community composition hence the two communities might have had different adaptation potentials to fluctuating pH. The Bergen community was dominated by diatoms, with some chlorophytes, cryptophytes and haptophytes, reflecting composition more common during the spring bloom^28^. On the other hand, the community in Svalbard was typical for post-spring bloom conditions and included haptophytes, prasinophytes and only minor contributions of diatoms. Here, dinoflagellates also became significant contributors during Bloom 2 and Bloom 3^27^.

The increase of labile DOC in form of DAA and possibly other bioavailable molecules also fueled growth of the microbial communities in our study. This was visible in the increased concentrations of PAA, especially during the post-bloom phases in the Raunefjord/Bergen campaign. Assuming that PAA represents heterotrophic and autotrophic microorganisms our findings are in line with results from previous publications. Endres et al.^25^ detected an 11–21% increase in bacterial biomass in high *p*CO_2_ mesocosms during Post-Bloom 1 and a 28% increase during Post-Bloom 2. Interestingly, the response in PAA is linear and does not show a pH threshold where the relationship reverses. This may be due to a better pH tolerance of extracellular enzymes used by heterotrophs, which show a higher efficiency under low pH conditions^25,46^. The lack of a clear signal in PAA in the Kongsfjorden/Svalbard campaign could be explained by the absence of increased bacterial biomass under elevated *p*CO_2_^47^. During this campaign bacterial numbers actually decreased in high *p*CO_2_ mesocosms and it was hypothesized that increased abundance of viruses led to higher levels of viral lysis resulting in a top-down control of bacterial biomass^47^, which however did not cause shifts in the bacterial community composition^48^.

Furthermore, gel-like particles may also play a role in fueling heterotrophic communities. TEP formation increased by 5–10% in high *p*CO_2_ mesocosms in Bergen under nutrient limited conditions^25^. While increased bacterial abundance is reflected by increased relative concentrations of PAA, increased TEP contribution is not easily detected within our TCHO. Increased contributions of TEP to TCHO would be indicated by elevated contributions of acidic sugars^21^. We would have expected elevated concentrations or increased relative contributions of e.g. GlcA, GalA or GlcUA to reflect a build-up in TEP. During the Raunefjord/Bergen campaign TEP peaked at the end of bloom phase 1 and 2^25^, however no detectable changes occurred in the CHO composition (Fig. 6). On the other hand, these TEP-related CHO showed enrichment during Bloom 3 in Kongsfjorden, indicating that TEP may have been increasingly abundant there. It therefore seems to be important to determine changes in TEP concentrations separately and not infer TEP abundance from changes in biomolecule composition.

The increase in DOC released from primary production under future ocean conditions has been described before^3,30^. Concurrently, this seems to also result in an increase in semi-labile compounds especially DAA and some DCHO. Contributions of % DAA to DOC were significant for some mesocosms in Kongsfjorden/Svalbard and for 7 out of 8 in Raunefjord/Bergen (p < 0.05). Here, changes were also correlated with *p*CO_2_ during two bloom phases (Bloom 1 in Raunefjord/Bergen and Bloom 2 in Kongsfjorden/Svalbard, Fig. S2). On the contrary, contributions of % DCHO to DOC only showed significant increased contributions in two mesocosms in Kongsfjorden/Svalbard (*p*CO_2_ = 890, 3045 µatm, p < 0.05) and control mechanisms should be investigated further (Fig. S2).

Potential consequences of increased contributions of semi-labile DOC with increasing *p*CO_2_ would stimulate the microbial carbon pump. Freshly produced DOC is quickly taken up, respired and reworked by bacteria leaving behind degraded and less bioavailable DOC that eventually will become part of the refractory organic matter pool^49,50^. Remineralization processes will enhance bacterial production, respiration and have a negative feedback on *p*CO_2_^30,31^. The refractory carbon left behind will in turn be transported to greater depths and stored in deeper ocean waters thereby sequestering atmospheric CO_2_^51^.

Under future ocean conditions, enhanced uptake of organic compounds and bacterial activity may also lead to increased competition for inorganic nutrients between autotrophs and heterotrophs with bacteria winning since heterotrophic bacteria are generally more successful in acquiring inorganic nutrients^52^. However, phytoplankton communities in low pH mesocosms shifted towards smaller sized phytoplankton^28,47^. Smaller species seem to be more tolerable to lower pH compared to larger species and benefit from a larger surface to volume ratios to effectively compete for inorganic nutrients^53^. High bacterial biomass may also be an advantage for mixotrophic protists, phytoplankton capable of bacterivory. In Kongsfjorden/Svalbard, numbers of dinoflagellates (a group containing mixotrophic species) positively correlated with elevated *p*CO_2_ during Bloom 2 and Bloom 3^27,54^ hinting that they might use bacteria as a food source. However, their top-down impact on bacterial biomass in high *p*CO_2_ mesocosms can only be speculated about. Additionally, a recent study by Anderson et al.^55^ showed that 40% and more of smaller phytoplankton (< 20 µm) also exhibited signs of bacteriovory, suggesting that this lifestyle might become more prominent under elevated *p*CO_2_. Hence more data is needed to determine if autotrophic biomass production and activity will be reduced in the future ocean as postulated by Thingstad et al.^56^.

Phytoplankton produces many AA and fatty acids that are essential to higher trophic levels. An increase in essential PAA contribution at the end of both campaigns (Fig. 4B,D) hint that the microbial biomass contained sufficient essential AA to be transported up the food chain to higher trophic levels. Essential FA content on the other hand diminishes under elevated *p*CO_2_^57^ and a smaller sized community decreases energy transfer efficiencies because more trophic intermediates are required to reach the highest trophic levels, reducing trophic transfer efficiency. This means that the verdict is still out on the extent of effects on food web interactions.

## Material and methods

### Experimental set-up of mesocosms and bloom development

Two mesocosm studies were conducted to investigate the effect of *p*CO_2_ changes on Arctic and Sub-Arctic marine ecosystems. In total, nine 25 m-long, free-floating Kiel Off-Shore Mesocosms for Future Ocean Simulations (KOSMOS) with flexible thermoplastic Polyurethane bags (∼ 50 m^3^) were deployed in Kongsfjorden, Svalbard (78.93° N, 11.89° E), in June–July 2010 and again in the Raunefjord near Bergen, Norway in April–June 2011 (60.31° N, 5.16° E). In both campaigns, a CO_2_ gradient was applied with eight different CO_2_ levels, only duplicating ambient CO_2_ conditions (control treatment without CO_2_ addition)^28,58^. Initial *p*CO_2_ levels were achieved by adding CO_2_ enriched seawater at different quantities. Samples for all parameters were taken with a depth-integrating water sampler (Hydrobios Kiel, Germany) for 1–15 m and 1–23 m, respectively. Daily vertical profiles were taken with a hand-operated CTD. Nutrient additions were performed to simulate upwelling of nutrient rich water and to stimulate a second phytoplankton bloom.

#### Kongsfjorden/Svalbard

Mesocosms were filled on 28 May 2010 with nutrient-poor, post-bloom fjord water (~ 50 m^3^ each). A 3 mm mesh size net was used to exclude large organisms. The bags were closed on 31 May 2010, defined as time t_−7_ and time steps (t) continued per day. The CO_2_ manipulation was done in steps over 5 days, from t_−1_ to t_4_, by adding calculated amounts of CO_2_ enriched seawater to 7 of the 9 mesocosm. Two did not receive additions and served as controls. Main CO_2_ additions were done from t_−1_ to t_2_ and a final adjustment was done on t_4_. Control mesocosms had initial *p*CO_2_ levels of 185/pH ∼ 8.32. The other mesocosms had initial *p*CO_2_ levels between 270 and 1420 μatm/pH 8.18–7.51 (detailed pH dynamics are provided by Schulz et al.^27^). Carbon system measurements and calculations were published in Bellerby et al.^59^. In short, total inorganic carbon and total alkalinity were measured. Partial pressure of CO_2_ (*p*CO_2_) and pH on the total hydrogen scale were calculated using salinity, temperature, dissolved nutrients and applying them with the CO2SYS program. Detailed development of pH is shown in Fig. S3.

Initial nutrient concentrations in the fjord water were < 0.1 µM nitrate, 0.09 µM phosphate, 0.2 µM silicate and 0.7 µM ammonium^27^. Additional inorganic nutrients were added at t_13_ (5 μM nitrate, 0.32 μM phosphate, and 2.5 μM silicate) and the experiment was terminated at t_30_. The experimental period was divided into three phases by previous authors^27^ based on the applied perturbations and Chl *a* dynamics covering the onset and demise of phytoplankton blooms. “Bloom 1” occurred before nutrient addition (t_4–13_), “Bloom 2” occurred after nutrient addition until the 2nd Chl *a* minimum (t_14–21_) and “Bloom 3” was from the 2nd Chl *a* minimum until the end of the experiment (t_22–29_)^27^. No clear post-bloom phases (longer period of low Chl *a* concentrations) were observed during this campaign.

#### Raunefjord/Bergen

Mesocosms were filled and closed on 30 April 2011, defined as time t_−8_. Seven mesocosms were adjusted over 5 days to *p*CO_2_ target levels ~ 400 to 3000 µatm/pH 8.13–7.20 by stepwise addition of CO_2_ saturated seawater (detailed information are given in Schulz et al.^28^). The control mesocosm M2 (300 µatm/pH 8.14) was omitted from statistical analysis because a hole was discovered during the first week of the experiment which could not be mended and salinity changes confirmed that several cubic meter of fjord water were exchanged. Carbon system measurements and calculations were published in Schulz et al.^28^. In short, pH was measured spectrophotometrically and presented on the total hydrogen scale. Dissolved inorganic carbon (DIC) was measured coulometrically and pH and DIC were used at in-situ temperature and salinitiy to calculate *p*CO_2_ and other carbonate system speciations. Detailed development of DIC and pH are shown in Fig. S4.

Initial concentrations of inorganic nutrients were higher than in the Svalbard campaign displaying 1.5 µM nitrate, 0.2 µM phosphate, 1.2 µM silicate and 0.45 µM ammonium^28^. The total added concentrations of nutrients at time t_14_ were 5 μM nitrate and 0.16 μM phosphate. No silicate was added to facilitate the growth of *Emiliania huxleyi*. The experiment was terminated at t_35_. Again, the experimental period was divided into four phases in previous publications^27,47^ based on the applied perturbations and Chl a dynamics. During “Bloom1” (t_3–8_) inorganic nutrients were rapidly taken up and lead to a first phytoplankton bloom. During the following “Post-Bloom1” (t_9–14_), there was little change in autotrophic biomass at low nitrate and phosphate availability. “Bloom 2” was after nutrient addition which initiated a second phytoplankton bloom (t_15–25_) and the “Post-Bloom2” (t_26–34_) showed again little change in autotrophic biomass, probably due to low phosphate availability.

We use the applied classification of bloom and post bloom phases to average our data.

A detailed description of the experimental setup, its deployment, technical features and the sampling methods are described by Schulz et al.^27,28^ and references therein.

### Chlorophyll *a* and dissolved organic carbon and nitrogen

Chlorophyll *a* (Chl *a*) for the Raunefjord/Bergen experiment, as well as Chl *a*, dissolved organic carbon (DOC) and nitrogen (DON) data for the Kongsfjorden/Svalbard experiment have been published elsewhere^3,27,28,46^ and were either retrieved from Pangaea^60^ or provided by the authors. DOC and DON samples for Raunefjord/Bergen experiment were collected and analyzed as described by Engel et al.^3^, and references therein. In short, 20 ml of sample was filtered through combusted GF/F filters and collected in combusted glass ampules. Samples were acidified with 100 µL of 85% phosphoric acid, heat sealed and stored at 4 °C in the dark until analysis. DOC samples were analyzed using the high-temperature combustion method (TOC-VCSH, Shimadzu). Total Dissolved Nitrogen (TDN) was determined with the TNM-1detector on the Shimadzu analyzer, which combusts nitrogen to NO_x_. NO_x_, chemiluminesces when mixed with ozone and is then detected using a photomultiplier^61^. DON concentrations were calculated by subtracting values of dissolved inorganic nitrogen concentrations (nitrate, nitrite, and ammonium). Sampling frequency was daily.

### Total and dissolved AA composition

TAA and DAA were determined separately. For TAA, 5 mL of sample were filled into pre-combusted glass vials (8 h, 500 °C) and stored at  − 20 °C until analysis. Samples for DAA were additionally filtered through 0.45 µm Acrodisc syringe filters before storage. Analysis was performed according to Lindroth and Mopper^62^ and Dittmar et al.^63^, with some modifications. In short, 1 mL of sample and 1 mL of 30% hydrochloric acid (Merck, suprapure) were hydrolyzed in sealed ampoules at 100 °C for 20 h. The hydrolysate was dried in a microwave at 60 °C under a nitrogen atmosphere and was washed twice with 0.5 mL of ultrapure water to remove any remaining acid. The final sample was re-dissolved in 1 mL ultrapure water for analysis. Analysis was performed on a 1260 HPLC system (Agilent) equipped with a C18 column (Phenomenex Kinetex, 2.6 µm, 150 × 4.6 mm) that can separate thirteen different AA after in-line derivatization with o-phtaldialdehyde and mercaptoethanol. Solvent A was 5% Acetonitrile (LiChrosolv, Merck, HPLC gradient grade) in Sodiumdihydrogenphospate (Merck, suprapur) Buffer (pH 7.0) and Solvent B was Acetonitrile, from 100% solvent A to 78% solvent A in 50 min. The detection limit for individual AA was 2 nmol monomer/L. The precision was < 5%, estimated as the standard deviation of replicate measurements divided by the mean. The following standards were used: asparagine + aspartic acid (AsX), glutamine + glutamic acid (GlX), serine (Ser), glycine (Gly), threonine (Thr), arginine (Arg), alanine (Ala), tyrosine (Tyr), valine (Val), isoleucine (Ile), phenylalanine (Phe), and leucine (Leu). γ-Aminobutyric acid (GABA) concentrations were below the detection limit in DAAs of Kongsfjorden/Svalbard mesocosms.

PAA concentrations were calculated: PAA = TAA − DAA. For a few individual AA in selected samples this resulted in negative values (DAA concentrations ≥ TAA concentrations) and values were omitted from the analysis. Throughout the Kongsfjorden/Svalbard dataset Gly in DAA was higher in concentrations than in TAA giving negative results and therefore Gly was omitted from the PAA fraction. This was probably due to a problem with the Gly standard during TAA analysis. Sampling frequency was every other day to keep sample number manageable during analysis.

### Total and dissolved CHO composition

TCHO and DCHO > 1 kDa were determined separately. Therefore, 20 mL were filled into pre-combusted glass vials (8 h, 500 °C) and kept frozen at − 20 °C until analysis. Samples for DCHO were additionally filtered through 0.45 µm Acrodisc syringe filters before storage. The analysis was conducted according to Engel and Händel^64^. First, samples were desalinated by membrane dialysis (1 kDa MWCO, Spectra Por) for 5 h at 1 °C, hydrolyzed for 20 h at 100 °C with 0.4 M HCl, final concentration, and then neutralized through acid evaporation under vacuum and nitrogen atmosphere (1 h, 60 °C). Samples were analyzed using high performance anion exchange chromatography coupled with pulsed amperometric detection (HPAEC-PAD) on a Dionex ICS 3000. The system was calibrated with a mixed sugar standard solution including the neutral sugars: fucose (Fuc), rhamnose (Rha), arabinose (Ara), galactose (Gal), xylose/mannose (Xyl/Man) and glucose (Glc), the amino sugars: galactosamine (GalN), glucosamine (GlcN), and the acidic sugars: galacturonic acid (GalUA), gluconic acid (GluA), glucuronic acid (GlcUA) and muramic acid (MurA). Particulate CHO concentrations were not determined, because DCHO and TCHO concentrations were very similar or equal, which resulted in negative particulate CHO concentrations for several of the data points throughout the dataset. When differences between DCHO and TCHO are small, these negative values can occur for two reasons. Firstly, during dialysis some TCHO can escape through the membrane (> 1 kDa) and affect final concentrations. Secondly, we run DCHO and TCHO batches separately (instead of DCHO sample followed by TCHO of same sampling point). Maintenance of the HPAEC-PAD system and exchange of analytical columns could induce minor differences in the calculated final concentrations. Sampling frequency was every other day to keep sample number manageable during analysis.

### Data analysis and statistics

To identify *p*CO_2_ dependent trends in AA and CHO concentrations we applied the method described by Endres et al.^25^. In short, the daily deviation (AD_*i*_) of each mesocosm was calculated by subtracting observed concentration (*X*_*i*_) from the average concentration of all mesocosms ($\stackrel{-}{X}$) for each sampling day (*AD*_*i*_ = *X*_*i*_–$\stackrel{-}{X}$). These daily deviations were checked for normality by inspection of histograms and Q–Q diagrams. In order to get the mean deviations (*MD*) of the entire bloom phase, all daily deviations per bloom and post-bloom phase were averaged, respectively, according to $\frac{1}{N} {\sum }_{i=1}^{N}\left({AD}_{i}\right)$ with *N* being the number of sampling days. Especially for the bloom phases this is important, as the bloom peaks were not reached at the same day across all mesocosms. The mean deviations were tested against initial pH of the different mesocosms by linear regression. If linear regression did not show significance levels p ≤ 0.1, a binomial regression was performed and significance was tested. This allows detection of trends that have a significant linear regression for a more limited pH-range.

Principle component analysis (PCA) was performed to explore differences in individual AA and CHO composition between different Bloom/Post-Bloom phases. Data for the relative contribution (%) of individual AA and CHO concentrations to sum of AA and CHO concentrations (nmol L) was used. The package CRAN:factoMineR in the open source software R was used for the PCA analysis using a correlation matrix.

## Supplementary information

Supplementary information.

## Data Availability

Concentration data for DOC, DON, TAA; DAA; TCHO and DCHO are openly available through the Pangaea repository: https://doi.pangaea.de/10.1594/PANGAEA.920945 (Svalbard), https://doi.pangaea.de/10.1594/PANGAEA.921093 (Bergen). All other relevant data are available from the corresponding author upon request.
