# Supplementary material for: Ocean acidification modifies biomolecule composition in organic matter through complex interactions
Source: Sci Rep. 2020 Nov 26;10:20599. doi: 10.1038/s41598-020-77645-3 (PMC7692504; doi:10.1038/s41598-020-77645-3)
Supplement: Supplementary file 1 — Supplementary information. [file 41598_2020_77645_MOESM1_ESM.pdf]

Supplemental Information

Ocean acidification modifies biomolecule composition in organic matter through complex interactions

Julia Grosse, Sonja Endres, Anja Engel

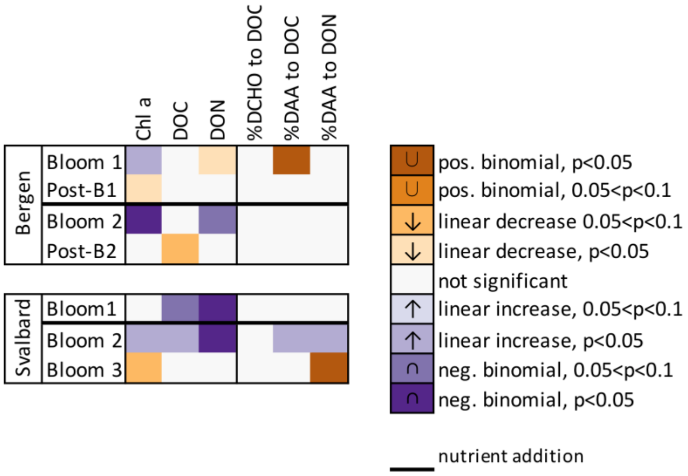

Fig. S1: Heat-map summarizes significance levels for organic matter parameters as calculated in Fig.1.

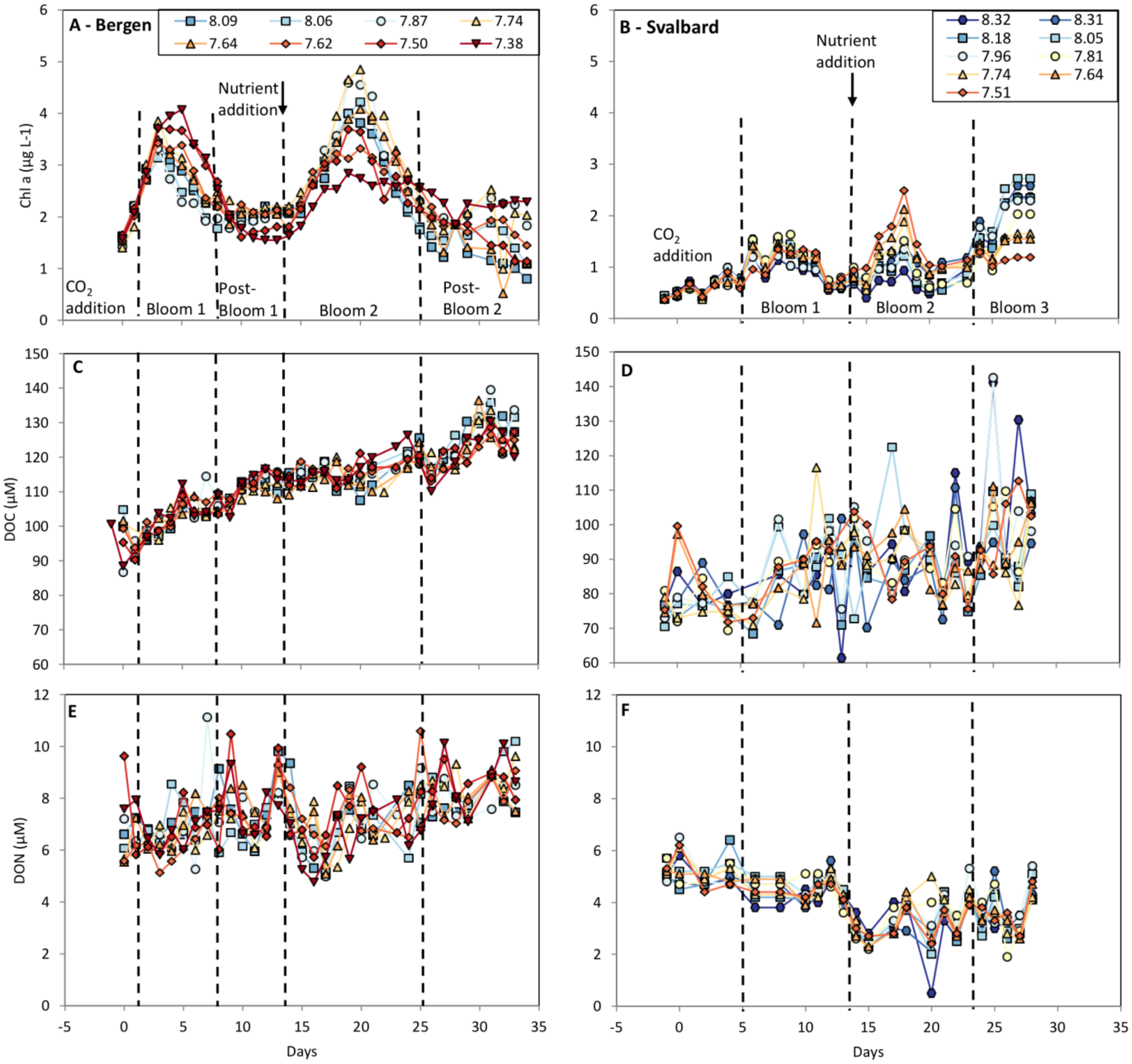

Fig. S2: Development of chlorophyll a, DOC and DON concentrations in all mesocosms in Raunefjord/ Bergen (A, C, E) and Kongsfjorden/ Svalbard (B, D, F). Shown are individual measurements in all mesocosms. The bloom phases and time point of nutrient additions are indicated as identified by Schulz et al. 2013, 2017. For symbol identification see also Tab 1.

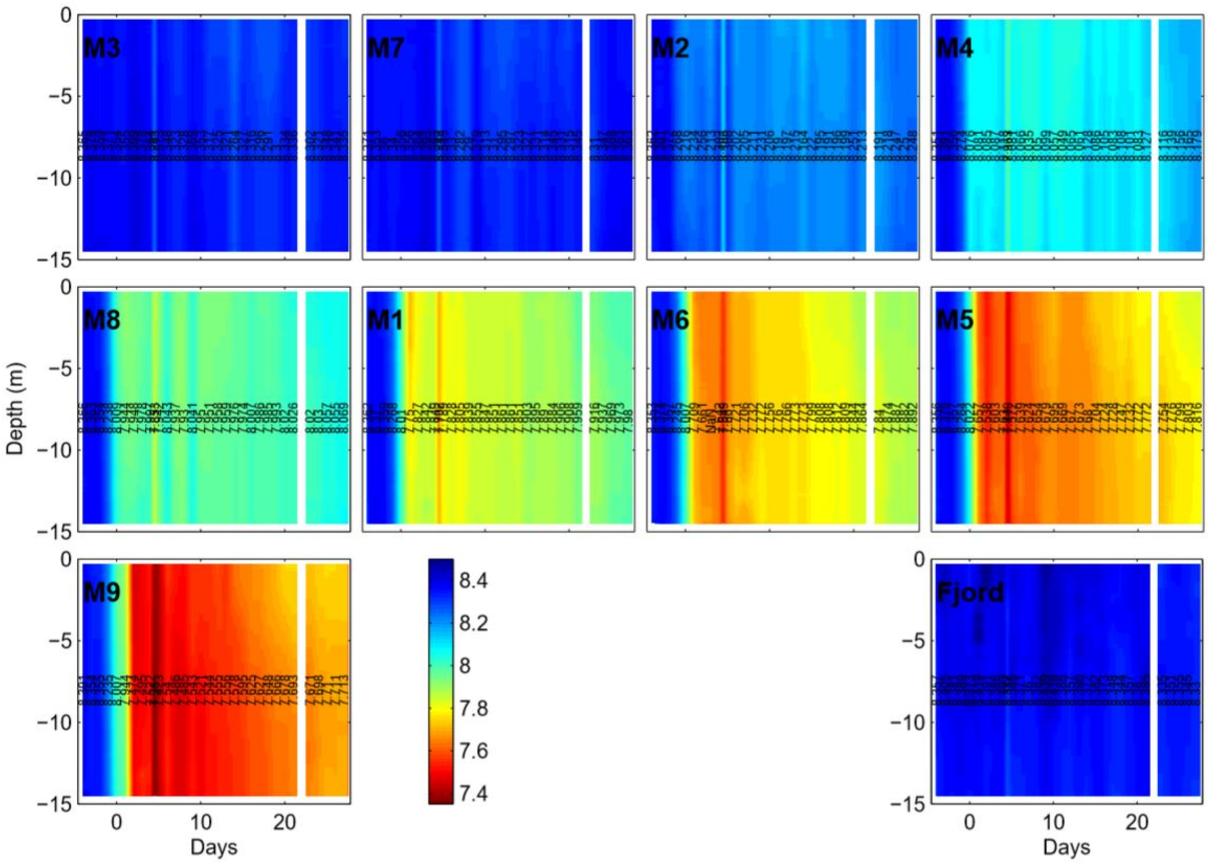

Fig. S3: Temporal pH dynamics in each of the Svalbard mesocosm and the fjord as published by Schulz et al. 2013. Recorded pH values were corrected by calculated pH from measured dissolved inorganic carbon and total alkalinity and are reported on the total scale. Black numbers denote daily depth-averaged (0.3–12m) mean pH values.

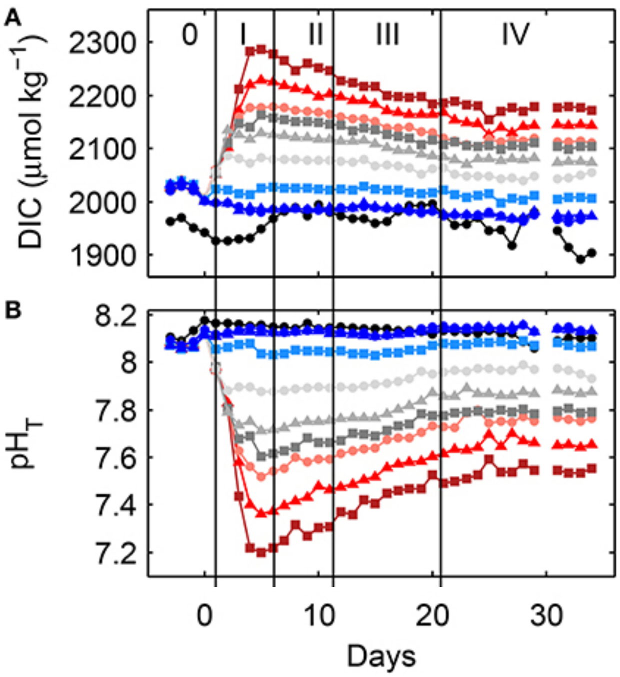

Fig. S4: Changes in depth-integrated (0–23 m) measured dissolved inorganic carbon (A) and pH_T (B) at in-situ conditions for Bergen mesocosms as published by Schulz et al. 2017. Vertical lines and Roman numbers illustrate the different phases during this experiment: 0 = CO_2 addition, I = Bloom 1, II = Post-Bloom 1, III = Bloom 2, IV = Post-Bloom 2.

Symbols and color refer to different mesocosms:

= pH 8.18 (fjord), = pH 8.14 (M2), = pH 8.13, = pH 8.04, = pH 7.88, = pH 7.71, = pH 7.60, =pH 7.52, = pH 7.36, = pH 7.20

References:

Schulz, K. G., Bellerby, R. G. J., Brussaard, C. P. D., Büdenbender, J., Czerny, J., Engel, A., Fischer, M., Koch-Klavsen, S., Krug, S. A., Lischka, S., Ludwig, A., Meyerhöfer, M., Nondal, G., Silyakova, A., Stuhr, A., and Riebesell, U. (2013). Temporal biomass dynamics of an Arctic plankton bloom in response to increasing levels of atmospheric carbon dioxide, Biogeosciences, 10, 161–180, https://doi.org/10.5194/bg-10-161-2013

Schulz KG, Bach LT, Bellerby RGJ, Bermúdez R, Büdenbender J, Boxhammer T, Czerny J, Engel A, Ludwig A, Meyerhöfer M, Larsen A, Paul AJ, Sswat M and Riebesell U (2017). Phytoplankton Blooms at Increasing Levels of Atmospheric Carbon Dioxide: Experimental Evidence for Negative Effects on Prymnesiophytes and Positive on Small Picoeukaryotes. *Front. Mar. Sci*. 4:64. doi: 10.3389/fmars.2017.00064
